# Health benefits of 'grow your own' food in urban areas: implications for contaminated land risk assessment and risk management?

**DOI:** 10.1186/1476-069X-8-S1-S6

**Published:** 2009-12-21

**Authors:** Jonathan R Leake, Andrew Adam-Bradford, Janette E Rigby

**Affiliations:** 1 Department of Animal and Plant Sciences, University of Sheffield, Sheffield S10 2TN, UK; 2 Department of Geography, University of Sheffield, Sheffield S10 2TN, UK; 3 National Centre for Geocomputation, National University of Ireland Maynooth, Co. Kildare, Ireland

## Abstract

Compelling evidence of major health benefits of fruit and vegetable consumption, physical activity, and outdoor interaction with 'greenspace' have emerged in the past decade - all of which combine to give major potential health benefits from 'grow-your-own' (GYO) in urban areas. However, neither current risk assessment models nor risk management strategies for GYO in allotments and gardens give any consideration to these health benefits, despite their potential often to more than fully compensate the risks. Although urban environments are more contaminated by heavy metals, arsenic, polyaromatic hydrocarbons and dioxins than most rural agricultural areas, evidence is lacking for adverse health outcomes of GYO in UK urban areas. Rarely do pollutants in GYO food exceed statutory limits set for commercial food, and few people obtain the majority of their food from GYO. In the UK, soil contamination thresholds triggering closure or remediation of allotment and garden sites are based on precautionary principles, generating 'scares' that may negatively impact public health disproportionately to the actual health risks of exposure to toxins through own-grown food. By contrast, the health benefits of GYO are a direct counterpoint to the escalating public health crisis of 'obesity and sloth' caused by eating an excess of saturated fats, inadequate consumption of fresh fruit and vegetables combined with a lack of exercise. These are now amongst the most important preventable causes of illness and death. The health and wider societal benefits of 'grow-your-own' thus reveal a major limitation in current risk assessment methodologies which, in only considering risks, are unable to predict whether GYO on particular sites will, overall, have positive, negative, or no net effects on human health. This highlights a more general need for a new generation of risk assessment tools that also predict *overall *consequences for health to more effectively guide risk management in our increasingly risk-averse culture.

## Introduction

In the UK there are estimated to be over 300,000 allotments [1] and in urban gardens fruit and vegetables are often grown in regions known to have a legacy of environmental pollution linked to former industrial activities, coal burning, motor vehicle emissions, waste incineration and dumping [1-3]. Cultivating and eating 'home-grown' foods holds both risks and benefits. Whilst local authorities and environmental protection agencies have focused considerable attention on assessment and management of the potential health risks of 'grow-your-own' (GYO) in urban areas, current risk assessment methodologies such as the Contaminated Land Exposure Assessment (CLEA) model [4] fail to consider the evidence of multiple health benefits of GYO, which may offset or more than fully compensate these risks. To date, an adequate synthesis of the *overall *risk to health arising from GYO in urban areas, has not been established. Without assessments of the overall risks to health it is not possible to effectively manage these overall risks. Risk managers need to be able to evaluate the relative risks and benefits to health in order to deliver a balanced and fully-informed strategy for management of overall health outcomes from GYO. This information needs to be provided from the risk-assessment process and in a form that allows meaningful comparisons between the risks and benefits. In contrast to this, current risk assessment models used in the context of GYO not only lack this holistic approach but may compound this limitation by using highly precautionary assumptions that often result in unnecessary contaminant 'scares' being instigated, and do not provide predictions of health outcomes that can easily be communicated to the general public. Whilst risk assessments under CLEA are cost effective as they follow a tiered approach with additional sampling and site investigations if contaminants exceed certain trigger thresholds, even these additional more accurate assessments of potential exposures to pollutants do not resolve the overall risks to health. As a consequence, expensive soil remediation treatments or site closures can occur where the risks to public health from pollutants are negligible and the benefits to health may be much greater than these risks.

## GYO- an antidote to sloth and obesity?

There is compelling evidence of major health benefits of fruit and vegetable consumption [5], physical activity, and outdoor interaction with 'greenspace' in urban areas [6]. Major physical, psychological and social benefits of GYO [7,8] have been demonstrated (Table 1). These health benefits, together with the environmental health risks associated with GYO in urban areas, need to be viewed in a wider context of public health risks. In recent decades significant environmental and societal changes appear to have shifted the balance of benefits and risks in GYO towards the benefits. The general population exposures to the heavy metals, polyaromatic hydrocarbons and dioxin pollutants that are considered the greatest risks to health have fallen in the UK since the 1980s, and this has been reflected in a 20-fold reduction in lead [9] and 71% decrease in dioxins [10] in commercially available foods in subsequent decades. Since most practitioners of GYO, including allotment holders with sizeable plots, still obtain the majority of their staple foods from shops [3], these changes indicate that the tolerable daily intakes of these contaminants from GYO are higher now than in the past two decades.

**Table 1:** Key potential health benefits of GYO in urban areas

Physiological
Multi-muscular exercise - improving cardiovascular function
Load bearing - reduced osteoporosis
Bending and stretching - increased general muscle tone
Outdoor exercise - 'fresh' air, sunshine
**Nutritional**
Fresh produce rich in vitamins and trace elements
Green leafy vegetables high in folic acid, iron and ascorbic acid
Brassicas (cabbage, cauliflower, broccoli, brussels sprouts, curly kale) rich in glucosinolates - implicated in preventing cancers
Legumes (peas, beans) are key components of the health protecting 'Mediterranean diet'
Berry fruits rich in anthocyanins, flavonoids and vitamin C
Apples rich in anti-oxidants implicated in cancer prevention
Sunlight exposure - leading to increased vitamin D synthesis in skin

**Psychological**
Sunlight exposure - increased serotonin (less winter-depression)
Sense of achievement and well-being - improved psychological health
Empowerment - independence/self sufficiency
Nature and greenspace interaction-increased well-being
Enhanced social networks and community interaction-increased well-being
Sense of community and belonging-increased well-being

(Adapted from Peraz-Vazquez et al. 2005).

However, these gains in environmental quality have been overshadowed by the rapidly escalating public health crisis of obesity linked to poor diet and lack of exercise. The lethal combination of 'gluttony and sloth' [11] resulting from excessive consumption of saturated fats and inadequate consumption of vegetables and fruit, together with increasingly sedentary lifestyles are amongst the most significant preventable contributors to cardiovascular disease, osteoporosis, diabetes and some cancers [11,12]. In the UK, there has been a 34% decrease in vegetable consumption in the past 50 years, one in five children eat virtually no fresh fruit or vegetables and 60% eat no leafy vegetables [12]. Nutritional deficiencies of iron and folic acid (both rich in leafy vegetables), Vitamin D (synthesised in skin from absorption of sunlight) and Vitamin C (rich in most fruit and vegetables) are implicated in poor health [13,14]. In low-income and materially deprived populations in the UK, it has been estimated that 25% of men and 16% of women have vitamin C deficiency [13] and approximately 50% of the elderly populations have sub-optimal Vitamin D concentrations [14]. Children today only spend half the amount of time outdoors compared to 20 years ago [12]. The time spent watching television, videos, and playing computer games has more than doubled compared to the time spent watching television in the 1960s, and these sedentary amusements contribute to inactive lifestyles and increasing childhood obesity [11].

Encouraging children from an early age to eat fresh fruit and vegetables has been shown to establish healthy choices and improve their long-term health [15]. This is likely to be further enhanced by active engagement of both children and adults in GYO providing outdoor exercise, and psychologically-enriching interaction with greenspace, together with the nutritional rewards of greater access to some of the key components of the life-extending 'Mediterranean diet'[16]. These include fresh leafy vegetables such as broccoli and other brassicas rich in glucosinolates, salads and legumes [16], together with berry fruits such as blackcurrants that are very rich in vitamin C and health-promoting flavonoids. Studies are now needed to establish whether involving children in GYO do indeed deliver these potential long-term health benefits.

## Health risks from urban pollutants in GYO?

In view of all these potential benefits, how great are the risks from environmental pollutants in GYO in urban environments? The major pathways of exposure to toxins through GYO are by ingestion of contaminated soil adhering to crops or through eating produce that has absorbed toxins from the soil [4]. Exposure to lead is the most important risk factor in typical urban areas [2,18], and has well documented adverse effects on health [18], although arsenic and polyaromatric hydrocarbons and a range of heavy metals may also present some risks [2]. The current method of risk assessment, CLEA, is based on measuring the concentrations of contaminants in soils, although lead, arsenic and polyaromatic hydrocarbons are not readily absorbed by food crops and their crop uptake is not reliably predicted from soil analysis [1,2,18] (Table 2). Furthermore, CLEA places considerable emphasis on soil ingestion [4] although the efficiency of toxin absorption by human digestion of soil is very poorly characterised, and soil ingestion is normally unintentional and unpredictable. Urban soils are normally enriched in 'black carbon' soots which strongly bind to most organic and inorganic pollutants inhibiting soil-to-plant pollutant transfers [17], and are likely to reduce the extent of gut absorption of soil-bound ingested toxins. For toxins present in fruit and vegetables our understanding of the extent of gut absorption is currently limited. There is therefore scope for further refinements of contaminant exposure risk models to increase their predictive power and reduce their reliance on precautionary assumptions which tend to inflate the estimated risks. Nonetheless, despite these limitations CLEA is currently the best available tool for assessing potential risks of exposures to soil contaminants.

**Table 2:** Lead concentrations in soil, vegetables and fruits in UK national surveys of agricultural soils and commercial foodstuffs, and in urban gardens and allotments, including two case study sites with high concentrations of lead in the soil.

Sampled area	Locations (UK)	Soil lead mg kg^-1 ^dwt				Vegetable lead mg kg^-1 ^fwt			References
	**Area**	**Mean/median**	**Range**	**n**	**Vegetable/fruit**	**Mean**	**Range**	**n**	**Soils/vegetables**

Agricultural soils	England & Wales Soil (5 km grid), Food from UK shops.	**74 **(Med)	NR	*5692*	Root vegetables Greens Potatoes Others	0.05 (M) 0.004 (M) 0.003 (M) 0.013 (M)	<0.05-**0.28** NR NR NR	*120* *24* *24* *24*	[2,20] [9] [9] [9]

Urban garden soils	50 cities, towns or villages.	**266 **(GM)	13-14100	*4127*	NR	NR	NR	NR	[2]

Urban allotment and vegetable plot soils	9 cities and towns.	**217 **(GM)	27-1676	*94*	Lettuce Spinach Cabbage Broccoli Carrot Parsnip	0.05 (GM) 0.14 (GM) 0.20 (GM) 0.21 (GM) 0.10 (GM) 0.13 (GM)	<0.02-**0.31** <0.02-**1.69** 0.06-**1.54** 0.06-**1.01** 0.02-**0.33** <0.02-**0.62**	*78* *80* *82* *82* *78* *84*	[19,19]

Urban allotment soils	6 cities	**464 **(M)	NR	*52*	Brassicas Legumes Potatoes Onions Soft Fruits All fruit and veg	0.008 (M) 0.009 (M) 0.009 (M) 0.007 (M) 0.017 (M) 0.010 (M)	NR NR NR NR NR <0.005-0.07	*68* *37* *72* *37* *36* *251*	[21,21]

Contaminated allotment soils (Case study 1)	Newcastle Walker Rd	**1200 **(M)	490-1900	*25*	Swede, turnip, beetroot, potatoes	<0.026 (M)	<0.001-**0.13**	*11*	[1,1]

Contaminated allotment soils (Case study 2)	London, location undisclosed	**1050 **(GM)	513-2910	*11*	Cabbage Carrots, parsnip, beetroot, potatoes	**1.83 **(M) **2.82 **(M)	0.18-**3.58** **0.87-5.35**	*4* *5*	[3,3]

GM= geometric mean, M= arithmetic mean, Med= median, n = the number of samples, NR = not reported, fwt = fresh weight. Figures in bold for vegetables indicate values in excess of current statutory limits [21] in commercially-produced food (0.1 mg kg^-1 ^fwt in potatoes and onions,0.2 mg kg^-1 ^fwt in small fruits and berries, 0.3 mg kg^-1 ^fwt in brassicas).

National surveys of lead concentrations in agricultural and urban soils show that the latter are typically more contaminated (Table 2). In a UK-wide risk assessment, the Food Standards Agency analysed crops from 6 urban allotment plots which have typical urban soil metal concentrations, and concluded that GYO posed no significant risks [20]. These findings accord with those of an earlier and larger study of 94 allotments and vegetable gardens in nine English towns and cities which found that although the geometric mean soil lead concentration was five times higher than in typical agricultural soils, less than 1% of 80 samples each of lettuce, spinach, broccoli, cabbage, carrot and parsnip exceeded the old statutory limit of 1 mg of lead kg^-1 ^fresh weight [20]. Risk assessment modelling of GYO for the West Midlands Conurbation in the UK [18], using a highly conservative risk index, indicated that food grown on 92% of the urban area presented minimal risk to the average person, although the subgroup of people consuming the largest amounts of own-grown produce in the most polluted areas were considered to have an increased risk - although the health outcomes of these risks were not quantified [18].

Two case studies, (Table 2), each involving a seriously contaminated urban allotment site, found no evidence of adverse health effects despite soil contaminant loadings much higher than typical urban sites [1-3,20]. These studies provide further reassurance that even at sites classed as badly contaminated the risks can be low. The first case, in Newcastle, involved a site with 200 plots in which major and widespread soil contamination by lead and arsenic together with local enrichment with polychlorinated dibenzo-dioxins and furans were reported [1]. Despite the soil contaminants considerably exceeding guideline values requiring intervention, minimal lead (Table 2), arsenic and dioxin contamination was found in vegetables and eggs produced from the site, and it was concluded that eating this produce would create no significant risk of harm. Nonetheless, because of the nature and extent of the soil contamination and the *apparent *risk suggested by the CLEA model, the site underwent expensive remediation treatment involving excavating and replacing the topsoil. The second case, in London, involved a site of 7 allotment plots with serious lead contamination [3]. Samples of cabbages and lettuce, and root crops (carrot, parsnip, beetroot and potato) revealed lead concentrations in most samples exceeding the new statutory limit set for commercial food (Table 2). However, none of the allotment holders reported suffering any chronic illness since working their plots, and some had rented their plots for up to 20 years. Analysis of blood-lead concentrations of a very small sample of allotment holders with contaminated plots revealed them to be in the normal range. Based on a worst-case scenario risk assessment, the site was closed down- the local authority approving remediation but 'the cost being prohibitive'[3]. The health risks from the stress, disturbance and loss of amenity to the plot holders in both these cases were not evaluated, but are likely to have caused considerable harm to wellbeing and mental health [22].

What is required in cases like these are for both the risks and benefits to health to be assessed in parallel using the same health outcome metric, so that their individual and overall net effects on human health can be determined to inform appropriate risk management. This can be built onto existing risk assessment tools such as CLEA through the development of predictions of the consequences of modelled pollutant exposures to health, together with parallel assessment of health benefits. One approach that allows this kind of overall synthesis of risks is the disability adjusted life years (DALY) assessment which expresses the number of life-years lost due to morbidity and mortality [23]. Refining a DALY like approach to a GYO context would enable negative effects of exposure to pollutants to be directly compared to health-promoting benefits of GYO, and overall risks calculated. Such an approach neither hides the specific risks nor ignores the health benefits, and provides the basis for effective risk management and for communicating and contextualising risks to GYO practitioners and statutory authorities.

## Conclusion

Growing your own food in urban areas has many potential health benefits which may positively improve the physical and psychological health of participants, and these health benefits may significantly offset or compensate for the apparently minor risks that follow from the higher loads of environmental pollutants in urban as compared to rural environments. The health benefits of GYO- which directly addresses some of the key national problems with diet and lifestyle- are likely to more than fully compensate risks at most sites that exceed current soil guideline values. This reveals a serious limitation in current risk assessment methodologies which, in only considering risks, are unable to assess to overall net effect of GYO on human health, and therefore may result in closure of sites that are providing significant overall health benefits to the GYO practitioners. This highlights the need to develop more sophisticated risk assessment tools that predict overall consequences for health from assessment of risk and benefits to health using a common metric and which can be built onto existing risk assessment tools such as CLEA. This will enable the individual and overall risks to health to be established to fully inform risk management decisions.

## Note

The peer review of this article can be found in Additional file 1.

## Competing interests

The authors declare that they have no competing interests. Both A. Adam-Bradford and J.R. Leake are urban GYO practitioners, each renting a council allotment plot.

## Authors' contributions

Jonathan Leake researched and wrote the paper, with assistance from Andrew-Adam-Bradford who undertook a literature review, with guidance and assistance from Jan Rigby.

## Supplementary Material

Additional file 1 Peer review. Click here for file
