# Supplementary material for: Health benefits of 'grow your own' food in urban areas: implications for contaminated land risk assessment and risk management?
Source: Environ Health. 2009 Dec 21;8(Suppl 1):S6. doi: 10.1186/1476-069X-8-S1-S6 (PMC2796502; doi:10.1186/1476-069X-8-S1-S6)
Supplement: Additional file 1 — Peer review. [file 1476-069X-8-S1-S6-S1.doc]

| Article ref no. | A1471-2105X SZ S6-S1 |
| --- | --- |
| Title | Health benefits of ‘grow your own’ food in urban areas: exposing a fundamental flaw in contaminated land risk assessments? |
| Author(s) | Jonathan R Leake, Andrew Adam-Bradford, and Janette E Rigby |

**Ref#1**

**General comments:**

This paper describes a potentially very interesting topic and raises some important issues. Unfortunately however, the paper in its current form is extremely unbalanced and comes across at an uninformed rant against the environmental risk assessment process so I recommend that it is not accepted for publication. I have listed my main comments below:

1. The authors are highly critical of the risk assessment process for soil contamination. They seem to ignore the fact that risk assessment is a tiered process so for example if the CLEA model indicates that there is a risk, then there are options for further risk refinement (e.g. through the use of bioaccessibility studies). It is not at all surprising that the CLEA model has identified risks where they don’t actually exist as approaches of this type tend to be highly conservative to address all the uncertainties that are present, it is only through higher tier studies that you can refine the risk assessment and reduce the uncertainties.
2. The authors seem to be mixing up risk assessment and risk management. In my opinion, cost-benefit analysis should not be incorporated into the risk characterization process as the authors seem to suggest (e.g. they suggest in the intro. that the CLEA model should consider the benefits), this is a risk management decision. It would have been more useful if the authors had put forward some ideas on how the risk and benefits can be weighed up.
3. The paper does really contain much substance.
4. A more critical and balanced review of some of the literature that is cited would be useful.
5. In sections there seems to be confusion of the chemical classes e.g. on p1 a dioxin is not a PAH.
6. Overall, I don’t think the problem is the risk assessment process but I think it is fair to say that the models and experimental approaches used for assessing contaminated land could be improved as well as our understanding of how soil contaminants are accumulated into plants and absorbed through the gut. These messages don’t come out of the paper at all.

**Ref#2**

**General comments:**

This paper addresses an interesting and highly topical environmental problem area. Unfortunately, the paper in its present form is not suitable for publication as it lacks sufficient depth on which to base a major criticism of current risk assessment methods.

**Ref#3**

CLEA is not meant to take account of the health benefits of GRO. It is there to assess the potential risks. I agree therefore that it is a risk management issue. I believe the authors have an interesting paper, but need to stop tying themselves in knots by pouring cold water on CLEA (you get the feeling they are on a mission). Instead they could say that current assessment of contaminated land does not take account of health benefits, list the benefits and propose how they could inform risk assessment in the future. It is something useful to say.

## Responses to referees’ comments

# Ref # 1 & 2

Whilst we disagree with some of the comments and opinions of the first reviewer they have nonetheless been extremely helpful in drawing our attention to aspects of the original manuscript that have now benefitted from very explicit clarification and adjustment.

The “fundamental flaw” in the original title implied that current contaminated land risk assessments are flawed. We recognize that this is not strictly true- the assessments of risk from contaminants are as good as we can currently achieve and whilst they have limitations (and scope for refinement) they are not fundamentally flawed in assessing the risk of pollutant exposure. However, the way in which these assessments have been used without any consideration of the health benefits of GYO is fundamentally flawed in terms of overall risk management. The overall risks and benefits to health need to be assessed - and the lack of the latter is a major limitation in current practice.

We have revised the title and the text accordingly to reflect these points.

These revisions also involve making clear that current risk assessment methods such as CLEA should not be abandoned but rather should be refined into predictors of health outcomes and augmented by parallel health benefit assessment tools that allow risks and benefits to health to be directly compared and overall effects on health to then be effectively managed.

Neither reviewer contests the facts and evidence we assemble in the paper, however the very negative perceptions of the paper expressed by the first reviewer appear to stem largely from their opinion that health benefits should not be considered in risk assessment but only in risk management. They perceive many of our criticisms of the risk assessment process to therefore be unjustified. This reflects current practice, and is no doubt a view that is widely held, but we remain completely convinced that effective management of risks requires the risks and benefits to be assessed in parallel and this information used to inform the risk management. We have now clarified and strengthened the arguments underpinning our case on this point, and in doing so explain briefly how risk and benefit can each be assessed using a directly comparable health metric.

We hope that you will find the revised version of the manuscript an insightful and significant contribution worthy of publication. Whilst we have been constrained by the length limits for this manuscript we manage to convey a lot of novel and important information in this concise format, and hope that the paper will be published so it can open the debate on how risks and benefits of GYO should be dealt with in the future.

# Ref # 3

We have carefully read through the paper again and given it to colleagues who are not involved in land contamination issues and we, and they, consider that the arguments we have laid out and the stance we have taken regarding the limitations in risk assessment of GYO are both reasonable and logical.

We have modified four sentences to further increase the clarity of our arguments and to improve the flow of the text, and added a reference to show the negative psychological impact of poor management of communicating these risks. However, as every one of the criticisms we have made of CLEA are all factually correct, fully justified, and none has been contradicted by any evidence from the reviewers we see no reason to remove any of them.

The limitations of CLEA are central to part of our argument justifying the development of a new holistic approach to risk assessment – as current dogma that health benefits should not be considered in risk assessment but only in risk management - is shown to lead to risk management decisions which may negatively impact health whilst being based on insecure, over-cautious and potentially biased assessments of the risks (e.g. assuming all contaminants in ingested soil are absorbed by the gut).

Unless there is a wider appreciation of the limitations of the approach used in CLEA nothing is going to change and we will continue to experience risk management of soil contamination in the context of GYO that exaggerates the actual risks, fails to provide a measure of likely health outcomes that is understandable by the public and policymakers and will discourage participation in GYO at a time when major expansion of GYO is highly desirable for public health, reducing greenhouse gas emissions, and increasing local food production in the UK. The present approach to contaminated land risk assessment is not best serving the interests of public health- and will need to substantially change if it is to do so.

In the absence of counter-arguments from the reviewers we defend our right to criticise the present approach, point out the limitations in current risk assessment methods, and to open the debate on how risks and benefits of GYO should be properly dealt with in the future.
